# Anesthesia Obtained by Use of Scopolamine with Reclus’ Method

**Published:** 1918-12

**Authors:** 


***Anesthesia Obtained by the Use of Scopolamine Together with Reclus' Method.*** By Dr. LAPEYRE. *Bulletin de la Société de Chirurgie de Paris,* May 28, 1918.

Dr. Lapeyre proceeds in the following way, when complete anesthesia might prove dangerous for the patient. He first injects scopolamine, which produces a partial unconsciousness, and then novocaine-adrenaline, which procures a localized anesthesia.

1/4 cgr. of morphia and 1/4 mgr. of scopolamine are injected an hour before operating. This is repeated half an hour before the operation. At the beginning of the operation, rapid anesthesia is obtained by means of novocaine.

1/2 mgr. of scopolamine is injected on the whole. It proves generally sufficient. 1 mgr. would be dangerous.

The surgeon operates as easily as if unconsciousness were complete.

Dr. Lapeyre made use of this method for the following operations: severe gastro-enterotomies impelled by cancers, intestinal resections, stangulated hernia with possible perforation of the bowel, amputation of a limb, prostatectomies; and, in the war-surgery for penetrating wounds of the chest or abdomen in shocked subjects, severe lesions of the limbs.

